# Current Advances in Multiple Myeloma: A Post International Myeloma Society (IMS 2022) Round Table Debate by the International Academy for Clinical Hematology (IACH)

**DOI:** 10.1007/s44228-023-00036-8

**Published:** 2023-04-15

**Authors:** Nizar J. Bahlis, Luciano J. Costa, Thierry Facon, Jean-Luc Harousseau, Salomon Manier, Aurore Perrot, Cyrille Touzeau, Mohamad Mohty

**Affiliations:** 1 University of Calgary, Calgary, AB Canada; 2 Division of Hematology/Oncology, O’Neal Comprehensive Cancer Center at University of Alabama at Birmingham, University of Alabama at Birmingham, Birmingham, AL USA; 3 University Hospital of Lille, University of Lille, Lille, France; 4 Institut de Cancerologie de l’Ouest, Centre René Gauducheau, Nantes-St Herblain, France; 5 Service d’Hématologie, CHU de Toulouse-Institut Universitaire du Cancer de Toulouse Oncopole, Toulouse, France; 6 Department of Hematology, University Hospital Hôtel-Dieu, Nantes, France; 7 Sorbonne University, Service d’Hématologie Clinique Et de Thérapie Cellulaire, Hôpital Saint Antoine, AP-HP, INSERM UMRs938, Paris, France

**Keywords:** Multiple myeloma, Immumotherapy, Stem cell transplantation

## Abstract

This round table discussion organized by the International Academy for Clinical Hematology (IACH) was dedicated to the 19th annual meeting of the International Myeloma Society (IMS), which was held in Los Angeles between the 25th and 27th August 2022. After some key meetings of the discipline of the field of clinical hematology, the IACH organizes regular round table discussion in order to summarize the flow of information and get the opinion of a panel of experts and the key take-home messages. As part of this discussion, the panellists debated 6 key topics: disease monitoring, management of high-risk multiple myeloma (MM), induction for newly-diagnosed MM, management of relapsed MM, immune reconstitution, and vaccination and cellular therapy in MM.

## Introduction

This round table discussion organized by the International Academy for Clinical Hematology (IACH) was dedicated to the 19th annual meeting of the International Myeloma Society (IMS), which was held in Los Angeles between the 25th and 27th August 2022. After every key meeting of the discipline of the field, the IACH organizes a round table discussion in order to summarize and to digest the flow of information and get the opinion of the panel of experts and the key take-home messages.

This discussion was moderated by Mohamad Mohty (MMo) from the Sorbonne University Hospital in Paris (France), joined by experts in this field: Aurore Perrot (AP) from the university hospital of Toulouse (France); Luciano Costa (LC) from the University of Alabama (USA); Salomon Manier (SM) from the University Hospital of Lille (France); Cyrille Touzeau (CT) from the University Hospital of Nantes (France); Thierry Façon (TF) from the University Hospital of Lille (France); Jean-Luc Harousseau (JLH) from the Cancer Center, University Hospital of Nantes (France); and Nizar Bahlis (NB) from the University of Calgary (Canada).

Six key topics were identified for the purpose of this discussion: disease monitoring; management of high-risk multiple myeloma (MM); induction for newly-diagnosed MM (NDMM); management of relapsed MM; immune reconstitution, vaccination and cellular therapy in MM.

## Disease Monitoring

SM: We have the IMWG criteria that were published in 2016 to assess the response and also the MRD of patients with MM, so we have two techniques approved by this recommendation. One is next-generation sequencing (NGS) for MRD assessment and the other is flow cytometry for disease monitoring. The depth of response at a sensitivity of 10^–5^ we can be improved up to 10^–6^, but sometimes it can be difficult for some patients and at 10^–5^ we can evaluate most of the patients. You have either a single time-point to evaluate MRD or a sustained emergent MRD and there are discussions about whether the time period should be over 6 or 12 months, but the IMWG recommended 12 months, with two time-points one year apart. Even when we have an emerging negativity in the bone marrow (BM) we can also have extramedullary disease (EMD) so PET-CT is becoming very useful in assessing emerging negativity and to ensure that you do not have EMD. We now have NGS or NGF (next generation flow) cytometry, PET-CT negative or positive MRD negativity. As you mention, we are more and more, looking for tools that we can use to look at blood samples instead of BM aspirates. The interest in mass spectrometry (MS) in the last few years is truly interesting; there are two different types we can use, for example, MALDI-TOF (matrix-assisted laser desorption ionisation time-of-flight), the easiest one to perform in the lab and the other is liquid chromatography-MS which is more complicated and needs more expertise. It is a very useful tool and results are close to what we can achieve with BM-NGS and flow cytometry. Perhaps it is a bit less sensitive with blood but we can repeat the time-points easily and so this MS method is something that is now being applied in clinical trials to evaluate the prognostic role of this tool for evaluation of MRD.

MMo: How is it on the other side of the Atlantic when it comes to monitoring MRD and whether you see added value in MS development?

LC: I would say in the US that as many of you know, a lot of the care of myeloma patients is carried out outside the academic institutions, essentially by general oncologists and not by myeloma experts and so the uptake of new diagnostics tends to be a little bit slow. There is a greater acceptance of BM-based MRD evaluation in academic centers but for the majority of patients treated in community hospitals, this is very uncommon. We are always hoping to refine prognostication, which is what these assays try to do, and I think it’s clear that MS is mostly (but not fully) overlapping with BM-based MRD. I think the value of that is likely going to be very context dependent. It’s essentially the detection of paraproteins at very low levels, I don’t expect it to be very useful early in disease treatment due to the very long clearance of the protein but I think we are going to refine and find a better use later on, both in identifying patients who still have disease and that can forego a BM-based therapy, and as we get better upfront therapy and create cohorts of patients who are observed without therapy, that might be one way to monitor disease resurgence short of full relapse that does not need frequent BM sampling. I think we should be reminded of the fact that BM-based MRD is where we have the most data and so we need to construct the use of MS without losing the anchor or reference to BM-based MRD.

MMo: Don’t you think that if we are able, at some point, to improve the threshold and sensitivity of MS, it can replace BM-based MRD because there is this invasive BM aspiration with how we measure MRD today?

CT: For sure MS is effective as a non-invasive method and the last data from the cytometric bead array test for instance, they reached almost a 10^–6^ sensitivity so I think it will be very important to test MS in the context of prospective trials to compare results with MRD-NGS. It’s a very attractive method. I think, we have all these prognostic factors, MRD-NGS, MRD-NGF, PET-CT and now MS data, the prognostic value is confirmed by prospective trials but what is the next step? Do we have to change our treatment based on MRD results from our patients, a question that I don’t think has been answered yet. We have prospective trials that are trying to answer this question and Luciano Costa presented the results of the MASTER [1] (Monoclonal Antibody-Based Sequential Therapy for Deep Remission in Multiple Myeloma, https://www.clinicaltrials.gov/show/NCT03224507) trial and we have the ongoing MIDAS [2] (MInimal Residual Disease Adapted Strategy, https://clinicaltrials.gov/show/NCT04934475) trial. For the first time I think we are trying to adapt patients’ treatment based on MRD which I think is the next step. We know the prognostic value of MRD, of sustained MRD and we have to move on in MM as for patients with diffuse large B cell lymphoma (DLBCL) or acute leukemia, MRD is a key test to change treatment.

MMo: Today, in your practice, how and when do you use MRD, how frequently do you use it, and if you use a PET scan, how frequently do you perform it?

AP: Today I use MRD monitoring for patients treated by autologous stem cell transplantation (ASCT), and I use both PET-CT for MRD outside the BM and NGS. We don’t use MRD to adapt treatment except in particular situations. For patients with high-risk MM, we sometimes use MRD to guide the second transplant or consolidation, but not for all patients.

MMo: In an elderly patient of 80 years old, do we need MRD monitoring or PET scans?

TF: I would say we need PET scans. I do not routinely use MRD in this particular patient group, but I would probably do a PET scan once per year. The way we assess bone is to do a PET scan at certain time-points. I think it’s fair to say that if you evaluate bone once per year, then it’s an acceptable approach in routine practice but with the DRd regimen (Daratumumab, Lenalidomide, Dexamethasone) you mention in an 80 year old, in the newly diagnosed patient I have to say that even if we achieve complete response which is the case for 50% of the patients, I do not see a very strong interest in looking at MRD and you could disagree with that but that is my clinical routine practice answer.

MMo: One of the key issues patients are struggling with is the continuous duration of therapy, its morbidity; so how can we handle this issue, assuming these techniques become available and easily feasible?

JLH: First, I’d like to comment on the MS issue. I think that currently, with the MALDI-TOF technology the sensitivity is not sufficient. I don’t think it is a usual tool for MRD assessment currently. The Spanish group has experience with flow cytometry showing that in the blood, it is possible to detect at a lower level of sensitivity, but if its positive, it’s not necessary to perform the BM assessment. At present, I think we are changing our minds on the interest in MRD assessment. Until now it was mostly useful for prognostication and now it is becoming a tool for deciding on treatment, but mostly within clinical trials. I appreciate LC’s comment about the real-life setting because in real-life we still don’t know how to use MRD assessment results to decide on treatment, to monitor, to tailor the treatment. It’s a question for clinical trials and yes, for clinical trials we have to use all of these MRD assessment tools repeatedly, PET scans in order to define the objective of the trial which is sustained MRD and then we have to decide if treatment can be continued or changed. The gain is mostly for clinical trials. I would like to finish by saying MRD assessment is useful if we have the objective of curing patients because for this, we need to achieve sustained MRD negativity. So, to repeat MRD assessment you need to have repeated PET scan negativity. I think that for a number of patients we can have very good results and progression-free survival (PFS) without obtaining MRD negativity, a lesson from the MAIA [3] trial, in which for elderly patients, the proportion of patients achieving MRD negativity was less than 30% and yet the PFS was more than 5 years. So, for such patients, maybe we don’t need to obtain MRD negativity but offer the best possible treatment and better quality of life than with aggressive treatment. We are trying to cure patients with more and more drugs, more and more expensive drugs, prolonging treatment duration and so very costly. Duration of treatment cannot be assessed without MRD assessment, but again, within clinical trials.

LC: I think MRD can be a bit of a divisive topic, but I think it is much easier to agree on how we are going to use MRD than to develop strategies for response adapted therapy because MRD is just a tool and at this moment it might be the best tool. The reality is that nearly 100% of the evidence that we rely on is based on treatments that we keep everyone on until they progress or complete a finite milestone. In reality we know that the response to therapy, whichever way you measure, it is the best indicator of how they are going to do, so it’s a shame that we don’t instrumentalise that more often.

## Management of High-Risk Multiple Myeloma

MMo: Let’s move now to the capacity of MRD negativity to overcome the baseline high-risk cytogenetic features or in other words, the dynamic assessment of the risk which brings me to the management of high-risk MM. What is the definition of high-risk MM for you today and what is the standard of care of these patients?

CT: To date, baseline cytogenetics is still a key prognostic factor and del(17p) and t(4;14) and chromosome 1 abnormalities are clearly associated with poor outcome but we learned from several prospective trials that MRD could overcome the impact of baseline cytogenetics. When we look at the IFM2009 [4] (Intergroupe Francophone du Myélome) trial we can see that patients with high-risk cytogenetics who achieved MRD negativity had a good outcome and it has been confirmed by the European Myeloma Network (EMN02) [5] trial that patients who achieved MRD negativity, even in the presence of adverse cytogenetics at baseline, had a good prognosis. I think prospective trials like the MIDAS [2] trial will answer key questions, but today we consider patients with del(17p), t(4;14) plus chromosome 1 abnormalities, as patients with adverse outcomes. For young patients, we could consider tandem transplant in addition to quadruplet induction and consolidation. For elderly patients, an anti-CD38 monoclonal antibody (mAb) plus lenalidomide and dexamethasone is the gold standard, even in the presence of adverse cytogenetics. I know some of us would consider quadruplet therapy for these high-risk patients, and we will have the results from two phase III trials, the CEPHEUS [6] trial (http://clinicaltrials.gov/show/NCT03652064) looking at quadruplet therapy, and IMROZ [7], evaluating the incorporation of the anti-CD38 mAb isatuximab (Isa) in elderly NDMM patients (http://clinicaltrials.gov/show/NCT03319667). I think the analysis of results of patients with high-risk cytogenetics will be very important.

MMo: So, what is the standard of care of the high-risk cytogenetics elderly MM patients who are fit and well?

TF: It is the so called DRd regimen. In fact, when you look at DRd, it has quite a strong benefit in terms of PFS compared to lenalidomide and dexamethasone. It also has some benefit in terms of overall survival (OS), so what would you like to do? Could you have another treatment proposal? I still recommend sticking to the DRd regimen. If patients are not frail, the question would likely be to use a combination of CD38 and bortezomib-lenalidomide-dexamethasone (VRd) if we are able to manage isatuximab or daratumumab combined with VRd and if the patient is able to tolerate this regime. I do believe this will be the best we could do for patients with high-risk cytogenetics before the availability of new immunotherapies.

JLH: TF just answered my question about the effect of daratumumab in high-risk cytogenetics in the MAIA [3] trial because the difference between DRd and Rd was less significant in high-risk patients, so probably DRd is not the top solution and if an elderly patient can tolerate daratumumab plus VRd, this may be more effective in these patients.

MMo: So, would you add a proteasome inhibitor (PI) for high-risk cytogenetics?

TF: We could speculate that ultimately it would be good, but we have to wait for the two major phase III studies with Isa or daratumumab in combination with VRd, IMROZ [6] and CEPHEUS [7]. When we have 1500 patients from these studies, we may be able to say that the benefit for patients with high-risk cytogenetics will be present or not, we don’t know, but if you can manage either daratumumab- or Isa-VRd, which is not the case for the majority of countries in fact, you may say that for an elderly fit patient, this regimen would be potentially the best treatment but we don’t have the data to support this presently.

MMo: Assuming we have a young and fit high-risk cytogenetics patient, most of the trials agree with the anti-CD38 mAb, but it looks to me that they tested carfilzomib-lenalidomide-dexamethasone (KRd) not VRd, what are your thoughts on this?

AP: I would say that for now it is not a recommended approach because there is no demonstration of superiority of carfilzomib over bortezomib especially in randomized studies and we can discuss the CLARION [8] (http://clinicaltrials.gov/show/NCT01818752) and ENDURANCE [9] (http://clinicaltrials.gov/show/NCT01863550) studies, but I think in the context of clinical trials that have an objective of adapting treatment based on MRD, we observe that carfilzomib-based regimens provide a deeper response and higher MRD negativity rates. This is why we use carfilzomib in that regimen but I’m sure that we can say today that we must use carfilzomib for high-risk patients.

MMo: What about the role of using novel immune therapies because this was debated during the IMS Congress, using for instance bispecific Antibodies for consolidation or even CAR T-cells as consolidation after a single ASCT.

SM: The trials are just starting and so it’s difficult to speculate on what would be the best. The assumption based on the biological point of view is that perhaps the immunotherapies will be less selective of the high-risk subclone of tumors than the conventional chemotherapies. Perhaps we can abrogate the poor prognosis of high-risk using immunotherapies. This is kind of hypothetical, and we need to see what happens with clinical trials. As has been mentioned there are several ongoing clinical trials that are evaluating CAR T-cells for example, instead of ASCT e.g. daratumumab-VRd followed by autologous CAR T-cells (ciltacabtagene autoleucel [cilta-cel] instead of daratumumab-VRd followed by ASCT (CARTITUDE-6 [10]) in frontline NDMM transplant eligible patients (http://clinicaltrials.gov/show/NCT05257083). There are trials [11], investigating the role of bispecific antibodies (BsAbs) in patients who don’t reach MRD negativity after ASCT and perhaps we should give them a little bit more than a simple lenalidomide maintenance. We need to see in the specific subgroup of high-risk patients whether there is an even better improvement in outcome in these patients because perhaps we will be able to destroy the high-risk clone as much as the standard-risk clones.

JLH: I would like to come back to the first question on the definition of high-risk. What is the role of defining risk? Firstly, for the patient and the doctor, to determine the overall prognosis. Secondly, compare trials and better evaluate new strategies and new drugs but also, it’s becoming more and more useful to tailor the treatment and I think that currently, it’s very difficult to tailor treatment if there is no consensus regarding the high-risk population. I hope that in the near future the IMS will succeed in organising a meeting of all the experts to at last define the group of high-risk patients. For the time being we have several stratifications, the simplest being the R-ISS [12] which is not Optimal; we have the French one [13], and the one developed by the EMN and perhaps the easiest system, the R2-ISS [14] with the three abnormalities that were mentioned above. I think it’s urgent to define high-risk cytogenetics patients and I would like to add one point which is that extramedullary disease is very important in defining the prognosis and finally, I’ll finish by saying that for the time being, since we are not able to reach a consensus regarding the definition of high-risk disease, the best way is to use the dynamic assessment and defining high-risk by the results of the induction treatments. In the MASTER trial [1], it was shown that ultra-high-risk (double hit) patients remain high-risk. I think a good way would be as in the MIDAS [2] trial and others, where they define high-risk by the response to very good induction treatment using modern quadruplet therapy. If a patient does not reach MRD negativity at the sensitivity of 10^–5^ he or she can be considered as high-risk whatever the cytogenetics; dynamic assessment of prognosis is becoming more and more important for defining the rest of the treatment.

LC: The issue of high-risk definition is complicated, if you talk to ten experts, you’ll get ten definitions and one thing you must be open to is the very notion of risk will change as therapies change. We have seen this in other diseases and things that were prognostic back in the days of melphalan-based therapy may no longer matter nowadays with quadruplet regimens. We saw in the CASTOR [15] trial (https://clinicaltrials.gov/ct2/show/NCT02136134) and some elegantly presented data from the FORTE [16] trial (http://clinicaltrials.gov/show/NCT02203643), where our Italian colleagues showed that in patients who become MRD negative, what seems to affect the risk of recurrence is whether they have two chromosome abnormalities *versus* one or zero. So, if we are going to split the population, the split may not be between standard- and high-risk any longer but between ultra-high-risk and the others, which is exactly what we saw in the MASTER [1] trial where patients with an isolated del(17p) or isolated t(4;14) did just as well as patients with standard-risk, including those who ceased therapy and went on without any maintenance. We are going to see the same data very soon from the GRIFFIN [17] trial (https://clinicaltrials/show/NCT02874742) where they split the patients into ultra-high-risk and high-risk. These patients are likely to be MRD positive at the end of induction, which we saw in MASTER [1] and I think we will see this in MIDAS [2] and others and so they are going to follow them as MRD positive at the end of induction where a different approach needs to be tried. It is not just a matter of continuing with the same therapy, we saw this in FORTE [16], they still do poorly and relapse despite continuing with double therapy. We saw this in GRIFFIN [17] as well but the data is still to be presented. So, what we are going to try to do in the near future is explore daratumumab-lenalidomide *versus* teclistamab-daratumumab as a consolidation in maintenance for these patients. The MIDAS [2] trial is going to help answer about double transplant and I really applaud initiatives looking at bispecific antibodies and CAR T-cells identifying that early combination of ultra-high-risk and the process of MRD positivity as the window of opportunity to test something new.

## Induction for Newly-Diagnosed MM (NDMM)

MMo: If you have a standard-risk cytogenetics MM patient with extramedullary localisation (EMD) at diagnosis, how would you handle this patient?

AP: I would not change my induction combination; I would use a quadruplet therapy with daratumumab-VRd outside clinical trials. I would monitor the EMD with PET-CT every 12 weeks to ensure that the response is not only of the biochemical component but also of the extramedullary disease and I will perform one or two transplants depending on the other high-risk characteristics. If there is only one EMD without high-risk cytogenetics and the patient has a good response I will consider only one transplant and then consolidation and maintenance. If the patient has other high-risk features, I will consider a double transplant.

MMo: There was some debate at the IMS Congress about bortezomib becoming a generic, lenalidomide is a generic; and the VRd combination has become affordable in many places. Thus, the debate is whether we should give VRd to non-transplant eligible patients and also to transplant eligible patients as induction, So, what is the role of VRd versus DRd?

TF: It seems that the cost of the VRd regimen with both the lenalidomide and bortezomib generics is about $10,000 per year which is very low compared with DRd, D-VMP (daratumumab with bortezomib, melphalan and prednisone) or even KRd and other regimens. You also have to keep in mind that the first-line is not the entire treatment, so for example, those patients who receive first-line VRd may receive CD38 in the second line, third line, and so will be exposed for a long period of time to CD38 antibodies as well. So, it’s not only the cost of first-line therapy. In the majority of countries, patients will receive either daratumumab or Isatuximab or both. So, if you don’t look at cost but look at efficacy, the median PFS for either VRd or KRd or even ixazomib and lenalidomide-dexamethasone is approximately 3 years and then when you look at DRd the median PFS is approximately 5 years so the benefit in PFS is about 2 years which is quite a long period for older patients. The efficacy of DRd is much better compared with VRd for older patients. We still don’t know with regard to OS, we expect the median OS with DRd to be approximately 7 years, we don’t have strong data for VRd in older patients, but from my point of view I would stick to the fact that we have to use the best option in front-line and if you have access to daratumumab in front-line I would go for DRd even though I know that there is a huge difference in terms of cost.

MMo: When do we use VRd in routine practice today because it looks like for the induction regimen in transplant eligible patients we switch to a quadruplet. D-VTd (daratumumab in combination with bortezomib, thalidomide, and dexamethasone) is approved, D-VRd is frequently used by many people and even D-KRd and Isa-KRd are becoming popular regimens.

CT: Today the only approved quadruplet induction for transplant eligible patients is D-VTd, based on the CASSIOPEIA [18] trial (http://clinicaltrials.gov/show/NCT02541383), the only phase III trial using a quadruplet combination. For sure, we have the results of the phase II trial GRIFFIN [17] in which the D-VRd arm is very attractive with a very good MRD negativity rate, excellent PFS and we are awaiting the results of the phase III PERSEUS [19] trial (http://clinicaltrials.gov/show/NCT03710603) which is testing D-VRd versus VRd in transplant eligible NDMM. So, for sure, D-VRd is very attractive and we use it outside of clinical trials in our daily practice, when you look at Grade 3–4 peripheral neuropathy (PN), there is no difference between D-VTd and D-VRd (CASSIOPEIA [18] versus GRIFFIN [17]). However, when you look at Grade 2 PN, and I think Grade 2 is very important for patients, D-VTd induces a higher rate of Grade 2 PN. I think that D-VRd will be the standard of care quadruplet induction for NDMM patients. I will talk about carfilzomib now and there are very interesting results for the carfilzomib quadruplet (Isa-KRd) from the German GMMG-CONCEPT [20] trial (http://clinicaltrials.gov/show/NCT03104842) dedicated to high-risk patients who received 6 cycles of Isa-KRd. The overall response rate was almost 100% and 60% achieved MRD negativity at the end of induction, so clearly KRd plus anti-CD38 is an excellent combination for this type of patient. We have also some results with KRd plus anti-CD38 and we have the EMN phase III IsKia [21] trial (http://clinicaltrials.gov/show/NCT04483739) of Isa-KRd versus KRd in transplant eligible NDMM patients which will probably show the superiority of Isa-KRd and maybe, in the next few months to a year we could have approval of front-line quadruplet with carfilzomib, but so far there is no such approval and so it is difficult to recommend.

## Management of Relapsed MM

MMo: Today, lenalidomide maintenance is being given to almost everyone whether transplant eligible or not. At the time of relapse, we are facing many patients who are lenalidomide refractory (Len-Ref), so what are the options and would be the role of the new immunomodulatory CELMoDs (cereblon E3 ligase modulators) such as iberdomide or mezigdomide in the Len-Ref patient? What is the eventual role of bispecifics for long-term maintenance, and whether CAR T-cell consolidation will allow us to stop maintenance?

SM: In patients with early Len-Ref relapse, we have some “old” options with daratumumab, bortezomib and dexamethasone (DVd) as in the CASTOR [15] trial, and carfilzomib and dexamethasone (Kd) as shown in the phase III ENDEAVOR [22] trial (http://clinicaltrials.gov/show/NCT01568866). These were the only options until recently. We now have more choices with the combination of an anti-CD38 and carfilzomib and dexamethasone as with carfilzomib, daratumumab and dexamethasone (KDd) as in the CANDOR [23] trial (http://clinicaltrials.gov/show/NCT03158688), and addition of Isa to Kd (Isa-Kd) showed significantly improved PFS and depth of response in the IKEMA [24] trial (http://clinicaltrials.gov/show/NCT03275285). Both of these trials offer new options but are there differences? It’s been discussed a lot, there will always be differences with different cohorts as they are not comparable exactly. The preferable option will be an anti-CD38 and Kd for those who are Len-Ref, but not anti-CD38 refractory of course. The question is do we need to switch the mechanism of action and so use a PI, or can we actually overcome the lenalidomide refractoriness with the new generation of IMiDs and use pomalidomide (Pd). We have the Pd, bortezomib and dexamethasone (PVd) approach from the OPTIMISMM [25] trial (https://clinicaltrials.gov/show/NCT01734928) which is a useful regimen. We have the results from the ICARIA [26] trial (http://clinicaltrials.gov/show/NCT02990338) showing that Isa-Pd demonstrated a significant improvement compared with Pd but this is for use after 2 prior lines of treatment, not at first relapse. So, it’s something that we know can overcome the lenalidomide refractoriness of patients. Then we have all the new options but we don’t have much data yet or approvals. We know that the CELMoDs iberdomide (CC-220; http://clinicaltrials.gov/show/NCT02773030) or mezigdomide (CC-92480) may perhaps overcome some lenalidomide refractoriness, they have a stronger affinity with cereblon and can potentially overcome some side effects based on the protein that will be degraded.

NB: The future therapies look very positive. We had a quick look at the data for CAR T-cell therapy from the CARTITUDE-2 [27] trial (http://clinicaltrials.gov/show/NCT04133636), cohort B, looking at Len-Ref patients who progress within 12 months. In the most recent update, cilta-cel gave a median PFS of over 70% at 2 years which seems to be superior to any other data we have seen with respect to the Len-Ref population. The other data on novel CELMoDs looks very promising and SM mentioned briefly the mezigdomide or CC-92480 data in patients who have multiple lines of therapies, up to four- or five-lines including Len-Ref, Pd and daratumumab refractory patients. We have seen an overall response rate of 50% in the phase I trial [28] (http://clinicaltrials.gov/show/NCT03374085) with extended data. The median PFS is still short in this population with mezigdomide-dexamethasone doublet therapy, but I can imagine that a combination with mAbs will look very promising. Lastly, BsAbs targeting BCMA, FcRH5, or GPRC5D, looks very promising in the highly-advanced relapsed population with a median PFS of around 12 months. For earlier MM relapsed patients, we have studies with teclistamab in the phase III MajesTEC-3 [29] trial (https://clinicaltrials.gov/show/NCT05083169) and elranatamab in the MagnetisMM-1 [30] trial (http://clinicaltrials.gov/show/NCT03269136). These studies have still to mature but I think we will see much more impressive durable responses than we have seen with the currently available regimens.

## Immune Reconstitution, Vaccination and Cellular Therapy in MM

MMo: Do you envision that the BsAbs and also the CAR T-cells will allow us to abandon the concept of continuous therapy and treat patients effectively with these agents?

JLH: There is a major difference between CAR-T cells and bispecifics in terms of duration of treatment. The former is one shot, possibly repeated, the latter treatment has to be continued. I’d like to ask the other experts an important question for me, is it possible to use BsAbs for a long period of time without increasing the exhaustion of the T-cell compartment? If the answer is ‘yes’ then OK, it will be possible to use BsAbs for a long period of time, if we don’t consider the cost of course. If the answer is ‘we don’t know’, we should be prudent and design trials comparing continuous therapy *versus* integral therapy and there is a paper in *Blood* regarding the T-cell exhaustion by BsAbs in B-cell precursor acute lymphoblastic leukemia [31], and it might be the same for MM, so I think that we have to learn more about the feasibility of long-term treatment with BsAbs before saying that it will be the solution in the long term.

LC: I couldn’t agree more and of course we don’t have the answer to that but when you have agents with that much activity as we see with BsAbs particularly in early lines of therapy, it begs the question whether patients need to be on it permanently. Some of these agents are designed to be of fixed duration and cevostamab is meant to be 1 year of therapy [32] (https://clinicaltrials.gov/show/NCT03275103). With continuous therapy, there can be loss of efficacy due to T cell exhaustion but you can also have the concerning issue of immunosuppression that predisposes to infection and hypogammaglobulinemia. So, I think there is a very strong argument to look for shorter durations of therapy.

AP: Regarding CAR T-cells I think they would be even more efficient if we use them in patients who haven’t been exposed to many drugs, so we are expecting very good efficacy in less advanced patients. We will see first, the results of KarMMa-3 [33] (https://clinicaltrials.gov/show/NCT03651128) and CARTITUDE-4 [34] (https://clinicaltrials.gov/show/NCT04181827) trials and in the KarMMa-3 press release we heard that idecabtagene vicleucel, the BCMA-directed CAR T- cell seems more efficient than standard regimens so we are waiting for these results and regarding the front-line, I think it will be very fast and we are very excited about the results of CAR T-cell therapy in the front-line. I don’t know if CAR T-cells can cure all patients and whether high-dose melphalan will disappear in the near future.

MMo: If you have a high-risk cytogenetics, young fit patient with del(17p), Del(1p) or 1q, when would you propose CAR T-cells?

NB: Immune therapy in principle should overcome high-risk disease driven by intracellular drivers like 17p deletion, so there is the premise of immune therapy. The data that we have so far for high-risk disease is a bit of a mixed bag. Looking at the forest plot for various CAR T-cell therapies, some high-risk studies show good results but the numbers are small, the hazard ratio interval is close to 1.0. CAR-T may improve the outcome of high-risk patients but not necessarily eliminate the poor outcome, which is really surprising to me because I thought that this would be one way that we could overcome the del(17p) and the t(4;14) also but the data doesn’t suggest a complete win. I think the future will be with some continuous therapy or continuous immune therapy in the sense that if we can vaccinate patients against their immune cells, and by vaccine, I mean as we do with allogeneic stem cell transplants, you induce cytotoxic T-cells to expand and persist and target different epitopes in these tumor cells and this will likely allow these patients to remain in remission for longer. We’ve seen some evidence of cytotoxic T-cell expansion with BsAbs as well as CAR T-cells, suggesting that this is one way of sustaining remission for longer.

JLH: I would like to raise again the issue of the role of CAR T-cells in the front-line and especially in the field of transplant eligible patients. The CARTITUDE-6 [10] trial (https://clinicaltrials.gov/show/NCT05257083), aims at replacing ASCT with CART-cells, but this does not solve the question of maintenance after CAR T-cells. Is it feasible, is it useful?

SM: We have some very preliminary data regarding this issue, but there are some trials, for example, KarMMa-7 [35]. Even though there is of course no label, some people are using it especially for patients who are not achieving MRD negativity after CAR T-cells and you perhaps need an extra boost so it seems to be doable. You need to wait for the patient to recover in terms of cytopenias so its somewhere around 3 months after CAR-T cell infusion that you can start maintenance treatment. Lenalidomide or pomalidomide might perhaps boost the CAR T-cells because they induce the expression or secretion of IL-2, so perhaps the CAR T-cell efficacy can be improved. We probably need randomised trials to see if it truly has an added value but it’s an interesting track and of course as mentioned, we need to think of fixed duration of treatments so perhaps we need to target a specific subset of patients, for example, those who don’t reach MRD negativity after CAR T-cells.

## Concluding Remarks

MMo: We have almost reached the end of this round table discussion. You have one minute each to give our audience, our colleagues, your take home message from the IMS Congress.

AP: I think the time has come to adapt to personalised treatment and especially to MRD.

CT: We have these great new therapies such as CAR-T and bispecifics and we still have to discover the mechanisms of resistance to such immune therapies. We have to understand the way myeloma cells escape from such immune therapies.

LC: In practical terms, there is greater acceptance of quadruplet therapy and greater optimism for response adapted therapy going forward. In terms of the future, I think it’s becoming clear that MM is more and more becoming an immunotherapy disease. Investigators have done a lot of work in the past in genomics of myeloma, mostly to assess the notion of risk for disease subtypes. The future technology, both in genomics and immune profiling, would allow to assign patients to a certain therapy, where you identify specific acquired alterations in the disease to choose therapy.

SM: One important communication at IMS was the one by Adam Cohen on CARTITUDE-2 [27], patients who relapsed and were previously exposed to BCMA targeted therapies and actually for the first time we saw that cilta-cel did not do so well in this cohort with a response rate of about 65%, with quite a short PFS. So, its perhaps not a very optimistic message but it seems that we have to really work on how to sequence those immunotherapies.

MMo: I think you are really highlighting the importance of real-world data and evidence because when you go outside clinical trials things become a little more different.

NB: There are multiple take home messages and LC mentioned the adaptive therapy which we need to start doing that more proactively and learn that one can start therapy based on MRD and incorporating into that, the high-risk features. The work on defining high-risk subgroups will be essential because I think high-risk definition will be dynamic based on the treatment we provide and the change in therapeutics that we provide. Understanding the mechanism of resistance to immunotherapy is going to be essential in allowing us to sequence these therapies and overcome the mechanisms of resistance. The highlight for me was really the preclinical work from the Spanish colleagues on the mouse model they have developed with the variable genetic backgrounds. Its going to be an amazing model, an immunocompromised model which will allow us to hopefully have answers to some of these questions we have on immune therapy in a preclinical model.

MMo: Obviously, if we can have a mouse model that can mimic the human MM, this will make the work much easier.

JLH: There were many important messages in each of the six topics but I’d like to focus on the key word of the meeting, which was ‘cure’. We were speaking of cure in each of the topics. Currently, the problem is we still don’t know how to define cure. Is it sustained MRD negativity, and for how long? Is it the possibility of stopping treatment and living without treatment? Or, on the contrary, is it living with the disease without MRD negativity? If you have a 74-year-old patient and you give him 10 years survival in good condition as a priority objective if not a cure. We are very encouraged by the fantastic improvements witnessed over the past 10 years and continue to see with immune therapy. We hope that a cure is possible, at least for standard-risk patients, but I think it would be interesting to think again about the definition of cure in MM.

MMo: Personally, I think cure is living long and well and all the rest is semantics. Thank you all for your wonderful contributions. We have tried, in less than 90 min, to summarise three very long, full days of a key congress. I hope we have managed to convey some important messages. Thank you.

## References

[1] Costa LJ, Chhabra S, Callander NS (2021). Daratumumab, carfilzomib, lenalidomide and dexamethasone (Dara-KRd), autologous transplantation and MRD response-adapted consolidation and treatment cessation. Final primary end point analysis of the Master trial. Blood.

[2] MInimal Residual Disease Adapted Strategy (MIDAS) KRd-Isatuximab: Frontline Therapy for Patients Eligible for Autologous Stem Cell Transplantation Less Than 66 Years; a Prospective Study https://clinicaltrials.gov/ct2/show/NCT04934475

[3] San-Miguel J, Avet-Loiseau H, Paiva B, Kumar S, Dimopoulos MA, Facon T (2022). Sustained minimal residual disease negativity in newly diagnosed multiple myeloma and the impact of daratumumab in MAIA and ALCYONE. Blood.

[4] Avet-Loiseau H, Lauwers-Cances V, Corre J, Moreau P, Attal M, Munshi NC (2017). Minimal residual disease in multiple myeloma: final analysis of the IFM2009 trial. Blood.

[5] Oliva S, Hofste op Bruinink D, ŘÍhová L, Spada S, van der Holt B, Troia R (2017). Minimal residual disease (MRD) monitoring by multiparameter flow cytometry (MFC) in newly diagnosed transplant eligible multiple myeloma (MM) patients: Results from the EMN02/HO95 phase 3 trial. J Clin Oncol.

[6] Zweegman S, Usmani SZ, Chastain K, Carey J, Ren K, Smith E (2019). Bortezomib, lenalidomide, and dexamethasone (VRd) ± daratumumab (DARA) in patients (pts) with newly diagnosed multiple myeloma (NDMM) for whom transplant is not planned as initial therapy: A multicenter, randomized, phase III study (CEPHEUS). J Clin Oncol.

[7] Orlowski RZ, Goldschmidt H, Cavo M, Martin TG, Paux G, Oprea C, Facon T (2018). Phase III (IMROZ) study design: Isatuximab plus bortezomib (V), lenalidomide (R), and dexamethasone (d) vs VRd in transplant-ineligible patients (pts) with newly diagnosed multiple myeloma (NDMM). J Clin Oncol.

[8] Facon T, Lee JH, Moreau P, Niesvizky R, Dimopoulos M, Hajek R (2019). Carfilzomib or bortezomib with melphalan-prednisone for transplant-ineligible patients with newly diagnosed multiple myeloma. Blood.

[9] Kumar SK, Jacobus SJ, Cohen AD, Weiss M, Callander N, Singh AK (2020). Carfilzomib or bortezomib in combination with lenalidomide and dexamethasone for patients with newly diagnosed multiple myeloma without intention for immediate autologous stem-cell transplantation (ENDURANCE): a multicentre, open-label, phase 3, randomised, controlled trial. Lancet Oncol.

[10] A study of daratumumab, bortezomib, lenalidomide and dexamethasone (DVRd) followed by ciltacabtagene autoleucel versus daratumumab, bortezomib, lenalidomide and dexamethasone (DVRd) followed by autologous stem cell transplant (ASCT) in participants with newly diagnosed multiple myeloma (CARTITUDE-6)

[11] ClinicalTrials.gov Identifier: NCT05317416. https://clinicaltrials.gov/ct2/show/NCT05317416

[12] Palumbo A, Avet-Loiseau H, Oliva S (2015). Revised international staging system for multiple myeloma: a report from International Myeloma Working Group. J Clin Oncol.

[13] Perrot A, Lauwers-Cances V, Tournay E, Hulin C, Chretien M-L, Royer B (2019). Development and validation of a cytogenetic prognostic index predicting survival in multiple myeloma. J Clin Oncol.

[14] D'Agostino M, Cairns DA, Lahuerta J, Wester R, Bertsch U, Waage A (2022). Second revision of the international staging system (R2-ISS) for overall survival in multiple myeloma: a European Myeloma Network (EMN) Report Within the HARMONY Project. J Clin Oncol.

[15] Weisel K, Spencer A, Lentzsch S, Avet-Loiseau H, Mark TM, Spick I (2020). Daratumumab, bortezomib, and dexamethasone in relapsed or refractory multiple myeloma: subgroup analysis of CASTOR based on cytogenetic risk. J Hematol Oncol.

[16] Gay F, Mina R, Rota-Scalabrini D, Galli M, Belotti A, Zamagni E (2021). Carfilzomib-based induction/consolidation with or without autologous transplant (ASCT) followed by lenalidomide (R) or carfilzomib-lenalidomide (KR) maintenance: Efficacy in high-risk patients. J Clin Oncol.

[17] Voorhees PM, Kaufman JL, Laubach J, Sborov DW, Reeves B, Rodriguez C (2020). Daratumumab, lenalidomide, bortezomib, and dexamethasone for transplant-eligible newly diagnosed multiple myeloma: the GRIFFIN trial. Blood.

[18] Moreau P, Attal M, Hulin C, Arnulf B, Belhadj K, Benboubker L (2019). Bortezomib, thalidomide, and dexamethasone with or without daratumumab before and after autologous stem-cell transplantation for newly diagnosed multiple myeloma (CASSIOPEIA): a randomised, open-label, phase 3 study. Lancet.

[19] Sonneveld P, Broijl A, Gay F, Boccadoro M, Einsele H, Blade J (2019). Bortezomib, lenalidomide, and dexamethasone (VRd) ± daratumumab (DARA) in patients (pts) with transplant-eligible (TE) newly diagnosed multiple myeloma (NDMM): a multicenter, randomized, phase III study (PERSEUS. J Clin Oncol.

[20] Leypoldt LB, Besemer B, Asemissen AM, Hänel M, Blau IW, Görner M (2022). Isatuximab, carfilzomib, lenalidomide, and dexamethasone (Isa-KRd) in front-line treatment of high-risk multiple myeloma: interim analysis of the GMMG-CONCEPT. Leukemia.

[21] Isa-KRd vs KRd in Newly Diagnosed Multiple Myeloma Patients Eligible for Autologous Stem Cell Transplantation (IsKia TRIAL) (IsKia) http://clinicaltrials.gov/show/NCT04483739

[22] Dimopoulos MA, Goldschmidt H, Niesvizky R, Joshua D, Chng W-J, Orio A (2017). Carfilzomib or bortezomib in relapsed or refractory multiple myeloma (ENDEAVOR): an interim overall survival analysis of an open-label, randomised, phase 3 trial. Lancet Oncol.

[23] Usmani SZ, Quach H, Mateos M-V, Landgren O, Leleu X, Siegel D (2022). Carfilzomib, dexamethasone, and daratumumab versus carfilzomib and dexamethasone for patients with relapsed or refractory multiple myeloma (CANDOR): updated outcomes from a randomised, multicentre, open-label, phase 3 study. Lancet Oncol.

[24] Moreau P, Dimopoulos M-A, Mikhael J, Yong K, Capra M, Facon T (2021). Isatuximab, carfilzomib, and dexamethasone in relapsed multiple myeloma (IKEMA): a multicentre, open-label, randomised phase 3 trial. Lancet.

[25] Dimopoulos M, Weisel K, Moreau P, Anderson LD, White D, San-Miguel J (2021). Pomalidomide, bortezomib, and dexamethasone for multiple myeloma previously treated with lenalidomide (OPTIMISMM): outcomes by prior treatment at first relapse. Leukemia.

[26] Richardson PG, Perrot A, San-Miguel JF, Beksac M, Spicka I, Leleu X (2021). Updates from ICARIA-MM, a phase 3 study of isatuximab (Isa) plus pomalidomide and low-dose dexamethasone (Pd) versus Pd in relapsed and refractory multiple myeloma (RRMM). J Clin Oncol.

[27] van de Donk NWCJ, Agha ME, Cohen AD, Cohen YC, Anguille S, Kerre T (2022). Biological correlative analyses and updated clinical data of ciltacabtagene autoleucel (cilta-cel), a BCMA-directed CAR-T cell therapy, in patients with multiple myeloma (MM) and early relapse after initial therapy: CARTITUDE-2, cohort B. J Clin Oncol.

[28] Richardson PG, Vangsted AJ, Ramasamy K, Trudel S, Martínez J, Mateos M-V (2020). First-in-human phase I study of the novel CELMoD agent CC-92480 combined with dexamethasone (DEX) in patients (pts) with relapsed/refractory multiple myeloma (RRMM). J Clin Oncol.

[29] Mateos M-V, Bahlis NJ, Costa LJ, Perrot A, Pei L, Rubin ML (2022). Randomized, phase 3 study of teclistamab plus daratumumab versus investigator’s choice of daratumumab, pomalidomide, and dexamethasone or daratumumab, bortezomib, and dexamethasone in patients with relapsed/refractory multiple myeloma MajesTEC-3. J Clin Oncol.

[30] Bahlis NJ, Raje NS, Costello C, Dholaria BR, Solh MM, Levy MY (2021). Efficacy and safety of elranatamab (PF-06863135), a B-cell maturation antigen (BCMA)-CD3 bispecific antibody, in patients with relapsed or refractory multiple myeloma (MM). J Clin Oncol.

[31] Philipp N, Kazerani M, Nicholls A, Vick B, Wulf J, Straub T (2022). T-cell exhaustion induced by continuous bispecific molecule exposure is ameliorated by treatment-free intervals. Blood.

[32] Lesokhin AM, Richter J, Trudel S, Cohen AD, Spencer A, Forsberg PA et al. 1924 Enduring responses after 1-year, fixed-duration cevostamab therapy in patients with relapsed/refractory multiple myeloma: early experience from a phase I study. In: 64th ASH Annual Meeting and Exposition, Saturday, December 10, 2022

[33] Delforge M, Baz RC, Cavo M, Callander NS, Ghobadi A, Rodriguez-Otero P et al. 2323 KarMMa-3: A Phase 3 Study of Idecabtagene Vicleucel (ide-cel, bb2121), a BCMA-Directed CAR T Cell Therapy Vs Standard Regimens in Relapsed and Refractory Multiple Myeloma. 64th ASH Annual Meeting and Exposition Sunday, December 6, 2020.

[34] Janssen Research and Development, LLC. A study comparing JNJ-68284528, a car-t therapy directed against b-cell maturation antigen (BCMA), versus pomalidomide, bortezomib and dexamethasone (PVd) or daratumumab, pomalidomide and dexamethasone (DPd) in participants with relapsed and lenalidomide-refractory multiple myeloma (CARTITUDE-4). In: ClinicalTrials.gov [Internet]. Bethesda (MD): National Library of Medicine (US). 2000- [cited 2022 March 10]. Available from: https://clinicaltrials.gov/ct2/show/NCT04181827. NLM Identifier: NCT04181827.

[35] Raje NS, Berdeja JG, Rodriguez-Otero P, Green DJ, Jagannath S, Lonial S (2021). KarMMa-7, a Phase 1/2, dose-finding and dose-expansion study of combination therapies with Idecabtagene Vicleucel (ide-cel, bb2121), a BCMA-directed CAR T cell therapy for relapsed/refractory multiple myeloma (RRMM). Blood.

[36] Touzeau C, Krishnan A, Moreau P, Perrot A, Usmani SZ, Manier S (2022). Evaluating teclistamab in patients with relapsed/refractory multiple myeloma following exposure to other B-cell maturation antigen (BCMA)-targeted agents. J Clin Oncol.

